# Health problems and the rural area

**DOI:** 10.11606/S1518-8787.20180520supl1ed

**Published:** 2018-09-13

**Authors:** Euclides Ayres de Castilho, Helen Gonçalves

**Affiliations:** I Universidade de São Paulo. Faculdade de Medicina . Departamento de Medicina Preventiva . São Paulo , SP , Brasil; II Universidade Federal de Pelotas . Faculdade de Medicina . Programa de Pós-Graduação em Epidemiologia . Pelotas , RS , Brasil

The Brazilian society, similar to what has been occurring in different contexts, is subjected to intense and extremely rapid changes in its demographic structure, especially in the process of inversion of the urban-rural distribution of its population. Last surveys ^1^ show that only 15% of the Brazilian population live in rural areas, which shows the reverse of the old existing trend, changed in the previous five decades. If this may represent a decrease in quantitative demand, which is represented by the volume of persons seeking health care, it does not mean eliminating the complexity of the existing mortality and morbidity situation and the observed epidemiological transition, with the persistence of endemic patterns, such as in the current expansion of yellow fever.

The health care and concern aimed at the rural population raise interest since the beginning of the 20th century, focused especially on rural endemic diseases ^2^ . However, they attract the commitment and work of few researchers. For example, we can highlight the original and unique contributions offered by Carlos Chagas on the disease that bears his name ^3^ and also the works of Samuel Barnsley Pessoa, who with his group sought to answer questions posed by Hochman ^4^: “How can we overcome the serious problems of Brazilian rural populations? How could public health reach the countryside?”. As expressed and emphasized by Scopinho ^5^ , studies expose the precariousness of the living conditions in addition to the work context to which they are subjected. The author attributes this to how production is organized in the agrarian environment, which is fundamentally based on the intensive and extensive monoculture that results in and becomes an indisputable source of distress and illness ^5^ . We highlight that the agricultural economy is predominant in many rural areas of the country, which turns into a problem the rural-urban dichotomy in the health field.

A search in the scientific production, although not exhaustive, indicates that this issue is insufficiently explored from the perspective of collective health. This supplement of the *Revista de Saúde Pública* , whose edition we had the honor to coordinate, brings important help in understanding and knowing the aspects of the health reality experienced in the rural environment. Although it brings information about an area located in Southern Brazil, which is also marked by important social inequalities even if it has distinctly better and more favorable conditions than the rest of the nation, its usefulness to its larger objectives is undeniable: establishment of knowledge about the problems of the rural population for the proposal of elements to define specific health policies and disclosure of appropriate methodological issues for the development of studies that respond to the uniqueness of the rural environment.

The reader has at their disposal a diverse list of topics, which cover the methodological aspects faced by the authors and the evident diversity in relation to the surveys done traditionally in the urban area. In addition, the reader can appreciate relevant topics such as quality of life, mental health, nutrition perspectives, and life habits, among which we can mention nutrition, physical activity, smoking, and alcohol consumption.

The *Revista de Saúde Pública* fulfills its role of disseminating original knowledge by providing an important contribution to the topic of health in rural areas, which demands a more intense exploration.

 

## References

[1] Instituto Brasileiro de Geografia e Estatística (2010). Censo demográfico 2010.

[2] Hochman G (2010). O sal como solução? Políticas de saúde e endemias rurais no Brasil (1940-1960). Sociologias.

[3] Goldbaum M, Barreto ML (2008). Commentary: the contribution and the example of Carlos Chagas. Int J Epidemiol.

[4] Hochman G (2015). Samuel Barnsley Pessoa e os determinantes sociais das endemias rurais. Cienc Saude Coletiva.

[5] Scopinho RA (2010). Condições de vida e saúde dos trabalhadores em assentamento rural. Cienc Saude Coletiva.

